# Acquisition and Analysis of Excised Neocortex from Pediatric Patients with Focal Cortical Dysplasia Using Mesoscale Diffusion MRI

**DOI:** 10.3390/diagnostics13091529

**Published:** 2023-04-24

**Authors:** Chandler Fountain, Harmanvir Ghuman, Michael Paldino, Mandeep Tamber, Ashok Panigrahy, Michel Modo

**Affiliations:** 1 Department of Radiology and Medical Imaging, University of Virginia Health System, 1215 Lee St, Chartlottesville, VA 22903, USA; 2 Department of Bioengineering, University of Pittsburgh, 302 Benedum Hall, 3700 O’Hara Street, Pititsburgh, PA 15260, USA; 3 McGowan Institute for Regenerative Medicine, University of Pittsburgh, 450 Technology Drive, Suite 300, Pittsburgh, PA 15219, USA; 4 Department of Radiology, University of Pittsburgh, PUH Suite E204, 200 Lothrop Street, Pittsburgh, PA 15213, USA; 5 Department of Neurological Surgery, University of Pittsburgh, 200 Lothrop Street, Suite B 400, Pittsburgh, PA 15213, USA; 6 Centre for the Neural Basis of Behavior, University of Pittsburgh and Carnegie Mellon University, 4074 Biomedical Science Tower 3, Pittsburgh, PA 15261, USA

**Keywords:** surgical resection, MRI, diffusion tensor imaging, tractography, focal cortical dysplasia, mesoscale

## Abstract

Non-invasive classification of focal cortical dysplasia (FCD) subtypes remains challenging from a radiology perspective. Quantitative imaging biomarkers (QIBs) have the potential to distinguish subtypes that lack pathognomonic features and might help in defining the extent of abnormal connectivity associated with each FCD subtype. A key motivation of diagnostic imaging is to improve the localization of a “lesion” that can guide the surgical resection of affected tissue, which is thought to cause seizures. Conversely, surgical resections to eliminate or reduce seizures provided unique opportunities to develop magnetic resonance imaging (MRI)-based QIBs by affording long scan times to evaluate multiple contrast mechanisms at the mesoscale (0.5 mm isotropic voxel dimensions). Using ex vivo hybrid diffusion tensor imaging on a 9.4 T MRI scanner, the grey to white matter ratio of scalar indices was lower in the resected middle temporal gyrus (MTG) of two neuropathologically confirmed cases of FCD compared to non-diseased control postmortem fixed temporal lobes. In contrast, fractional anisotropy was increased within FCD and also adjacent white matter tracts. Connectivity (streamlines/mm^3^) in the MTG was higher in FCD, suggesting that an altered connectivity at the lesion locus can potentially provide a tangible QIB to distinguish and characterize FCD abnormalities. However, as illustrated here, a major challenge for a robust tractographical comparison lies in the considerable differences in the ex vivo processing of bioptic and postmortem samples. Mesoscale diffusion MRI has the potential to better define and characterize epileptic tissues obtained from surgical resection to advance our understanding of disease etiology and treatment.

## 1. Introduction

Focal cortical dysplasia (FCD) is a common malformation of cortical brain development that represents a clinically important cause of medically intractable epilepsy. The microscopic abnormalities seen in FCD were first observed by Crome in 1957 [1] and described more fully by Taylor and colleagues in 1971 [2], noting unusual “congregations of large, bizarre neurons”. However, the pathophysiology of this neurological condition remains poorly understood and has been the subject of numerous international consensus meetings. A classification proposed by the International League Against Epilepsy (ILAE), defines three major types of FCD [3,4]. The most recent advances in FCD have primarily concerned Type II, with further characterization of electroencephalographic, radiographic, and clinical features. On clinical MRI, areas of type II FCD typically show blurring of the grey–white matter interface, focal cortical thickening, focal hyperintense signal on T_2_-weighted sequences, and occasionally the “transmantle” sign of a thin hyperintense signal spanning from the cortex to the underlying ventricle [5]. In contrast, type I FCD is associated with a weak blurring of white/grey matter, accompanied by increased T2/FLAIR and decreased T1 signal in subcortical white matter [6]. FCD type III incorporates cases that do not meet the criteria for type I or II and is typically associated with a principal lesion on MRI and cortical lamination abnormalities in histopathological evaluations [3,7].

Structural MRI can be unremarkable in patients with FCD. For example, in a series of 118 patients with histologically proven FCD type IIa or IIb, MRI was interpreted as normal in 21% of patients [8]. In an earlier, smaller series of 52 patients with FCD, MRI was unrevealing in 34% of patients [9]. Characterization of FCD on MRI has improved in recent years, as more advanced imaging has become clinically available. Advanced methods of particular interest include diffusion tensor imaging (DTI) and tractography. One of the most exciting DTI findings in FCD includes detection of dysplasia beyond the visualized lesion on conventional MRI [10,11]. Recent diffusion imaging techniques, such as diffusion kurtosis imaging (DKI) and Neurite Orientation Dispersion and Density Imaging (NODDI), offer the promise of even more accurate characterizations of tissue microstructure with the primary drawback being acquisition time [12,13]. Fiber tractography is increasingly being utilized in surgical planning to avoid eloquent white matter (WM) tracts adjacent to areas of dysplastic cortex [7,14], but so far has not improved the differential diagnosis of FCD subtypes. In patients with FCD in the frontal or occipital cortex, the volume of fiber bundles in the affected cortical areas was reduced compared to homologous contralateral control regions and mean FA in these fiber bundles was also reduced [15]. Regions proximal to the FCD locus have been found to exhibit abnormal functional connectivity [16], but are likely to also affect distal regions [17,18]. It is expected that the microstructural diffusion measurements and tractography of the dysplastic region, as well as peri-lesional cortical grey and white matter, will improve the characterization of neocortical tissue and guide the resection of affected areas [14].

Resected tissue from patients with FCD provides a unique opportunity to gain a better understanding of the neuropathological underpinnings of this condition, as well as to develop quantitative imaging biomarkers (QIBs) that can aid in the differential diagnosis of FCD subtypes [19,20]. The use of ex vivo samples affords extended imaging times that can achieve a mesoscale resolution (µm to mm scale) but also acquisition of multiple contrast methods, such as T_1_, T_2_, and diffusion imaging [21,22]. To date, these studies have focused on relating structural MRI measures with histopathology, mainly by achieving a high in plane resolution of 136 μm [19] and 46 μm [20] using thick slices (500 μm and 700 μm, respectively). Nevertheless, the relationship between computer-generated streamlines from diffusion MRI and the underlying axonal connectivity remains unclear [23]. Human axons range between 0.3 μm [24] and 20 μm in diameter [25], which is much smaller than typical voxel sizes used in MRI-based tractography. Scalar indices provide a summary static of diffusion measurement reporting on tissue microstructure [26]. Line profile analyses (LPA) of intensity gradients across tissues on MR images revealed cortical blurring and an inverse relationship between T_1_, T_2_ and T_2_* intensities with myelin, as detected by histology [19].

The current study aims to further extend those observations by investigating diffusion-based changes in the microstructure of excised tissue samples that included pathologically proven FCD in surrounding grey and white matter. To calculate diffusion scalar indices and perform tractography in these samples, a MR acquisition using isotropic voxels at the mesoscale is required to avoid distortions of the diffusion signal and adequately separate white and grey matter [27,28,29,30]. We have previously demonstrated that this approach can discern individual cell layers and afford the visualization of aberrant intrahippocampal connections in cases of mesial temporal lobe epilepsy [22,29]. The primary objective of this study was to acquire and analyze isotropic mesoscale diffusion MRI that would afford a tractographic analysis of surgically excised FCD tissues. We hypothesized here that mesoscale diffusion MRI would allow for identification of unique microstructural characteristics (both intrinsic to the lesion and the grey/white matter cortical border) and surrounding aberrant connectivity, all of which are commonly associated with pathologically proven FCD lesions.

## 2. Materials and Equipment

### 2.1. Patient Selection

**Figure 1:**
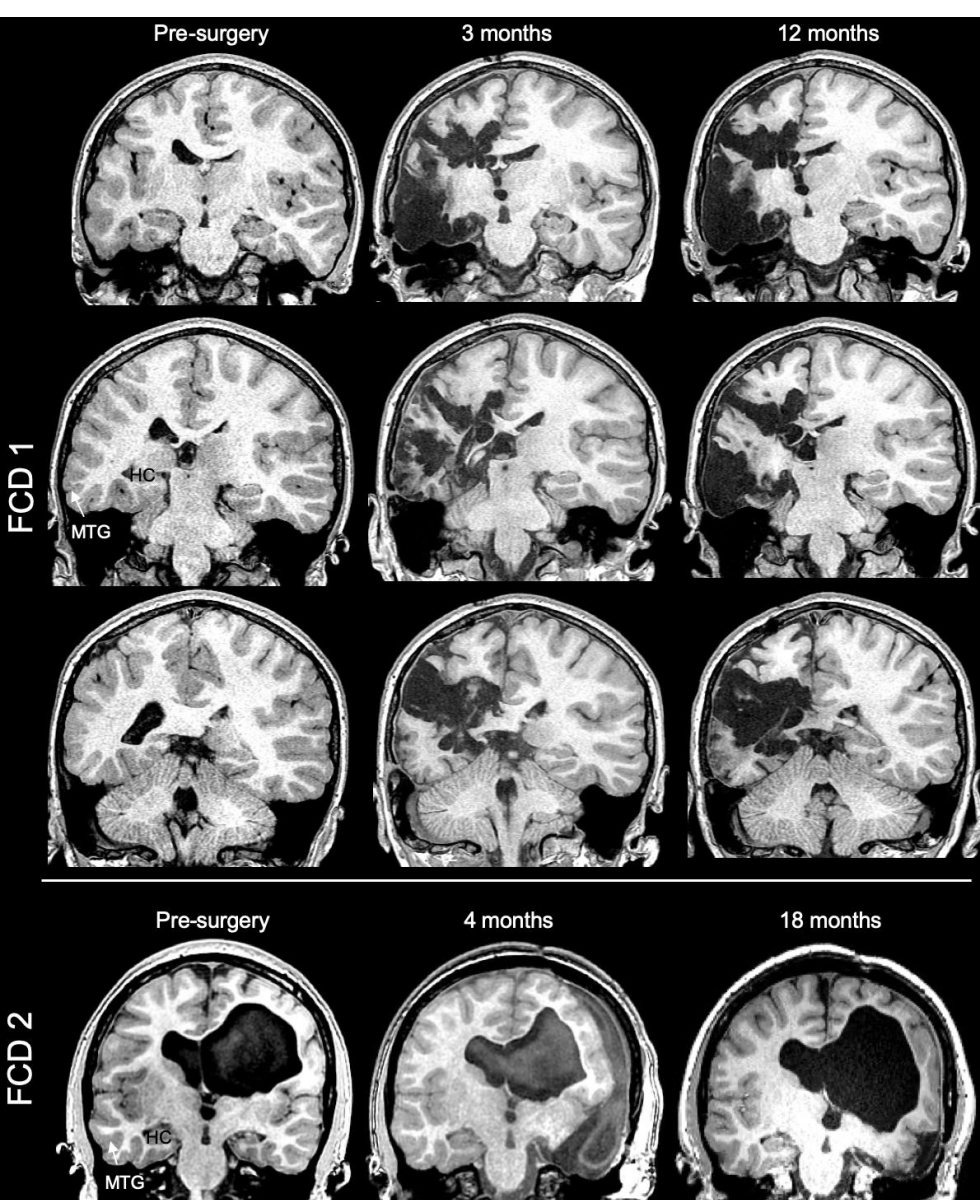
Pre- and post-surgery 3T MR imaging of both FCD cases. Extensive polymicrogyria was evident in extensive regions of the left hemisphere of FCD1, but especially the temporal lobe exhibited evidence of cortical malformation. In contrast, FCD2 experience a global injury during a premature birth that included hypointense subcortical white matter in the left hemisphere and periventricular hemorrhagic infarction in the right hemisphere that resulted in a hydrocephalus ex vacuo. Following case discussion by an interdisciplinary epilepsy surgical board, the subjects underwent extensive tissue resection of the suspected dysplastic regions driving seizure activity. FCD1 underwent a partial left hemispherectomy, whereas a right temporal lobectomy was recommended for FCD2. In both cases, surgical resection controlled seizure activity at 1 year post-surgery.

The protocol was approved by the Institutional Review Board in accordance with the Declaration of Helsinki (IRB; approval PRO11080392: Surgical Epilepsy Brain and Biomarker Databank) at the University of Pittsburgh, USA and complied with the Health Insurance Portability and Accountability Act (HIPAA) of 1996. Two subjects (8 and 15 years of age) with focal cortical dysplasia type I were confirmed by neuropathologic diagnosis. Informed consent from the patients’ parents was obtained to use resected materials for research purposes. All methods were performed in accordance with the IRB protocol, as well as guidelines and regulations pertaining to the use of human tissue samples. Preoperatively, the two subjects had pharmacoresistant epilepsy and a focus of suspected FCD within the temporal lobe, as demonstrated on clinical MRI imaging (Figure 1). Findings on preoperative 3T MRI included a combination of cortical thickening, blurring of the grey–white matter junction, and hyperintense T2 signal within the affected white matter. Following case discussion by an interdisciplinary epilepsy surgical board, the subjects underwent resection of their respective dysplastic focus. FCD1 presented with extensive polymicrogyria spread across most of the left hemisphere and underwent a peri-insular functional hemispherectomy to control seizure activity. FCD2 presented with a global injury at the time of prematurity, including the white matter, and periventricular hemorrhagic infarction prior to a left temporal lobectomy. Upon excision, brain tissue corresponding to the medial temporal gyrus (MTG) in the temporal lobe was immediately placed in 4% paraformaldehyde for fixation and was post-fixed for 48 h followed by storage in phosphate buffered saline (PBS) at 4°C. Only the MTG region was made available for research purposes. Both patients were seizure-free 1 year post-surgery.

Control specimens consisted of three postmortem temporal lobes from two different patients: patient 1 was a female donor aged 67 who died of multi-organ failure secondary to diabetes and hypotension (20 h postmortem delay to fixation); patient 2 was a male donor aged 63 who died of a pulmonary embolism secondary to obesity (19 h postmortem delay to fixation). Samples were obtained through the Committee for Oversight of Research and Clinical Training Involving Decedents (CORID, approval 384: The human connectome: from macroscale to nanoscale). Controls A and B represent the bilateral temporal lobes from patient 1 (averaged to provide single biological data point for the subject). Control C represents a unilateral temporal lobe from patient 2; the contralateral temporal lobe was not utilized. For control samples, postmortem tissue fixation occurred for 6 weeks in buffered formalin prior to transfer of samples to PBS. The physical dimensions for surgical (cortex only) and control specimen (whole temporal lobe) are listed in Table 1. Control samples were included to provide anatomical context for surgical samples [29].

### 2.2. MR Imaging

Imaging of the specimens was conducted on a 9.4 T Bruker Avance AV3 HD MR scanner equipped with a 72 mm diameter birdcage RF coil and Paravision 4.0 (Bruker Biospin, Billerica, MA). The specimens were vacuum-sealed in heavy-duty plastic sheets 24 h before being secured to the scanner bed using foam padding and adhesive tape to reduce scanner vibrations. Samples were consistently positioned using the same orientation with the cortical mantle oriented to the top of the scanner. The air temperature around tissue samples was maintained at room temperature (21 °C) by blowing ambient air into the magnet bore for the duration of scanning using a thermostatically coupled feedback system (Biopac, Goleta, CA). High-resolution anatomical images were obtained with a T_2_-weighted spin echo (SE) sequence, multi-slice-multi-echo (MSME) sequence, utilizing 30 equally spaced echoes (base TE = 8 ms, TR = 19,124 ms, NA = 3, isotropic resolution of 500 µm; 1 h 21 min scanning time). Diffusion imaging was obtained with a spin echo imaging sequence (directions = 6, TE = 28 ms, TR = 1100 ms, diffusion time = 15 ms; diffusion encoding = 4 ms, b-value = 6500 s/mm^2^, NA = 1, isotropic resolution of 500 µm; 15 h 30 min scanning time) to calculate scalar indices and afford high quality anatomical images. For tractography, a hybrid diffusion tensor imaging (hybrid DTI) 3D echo planar imaging (EPI) sequence was employed (TE = 22.24 ms; TR = 8000 ms; diffusion time = 15 ms; diffusion encoding = 4 ms; 3 shells at 1000; 4000; 10,000 s/mm^2^; directions = 30/shell, total = 90; NA = 1; isotropic resolution of 500 µm; 5 h scanning time). Five B0 scans were acquired using each sequence. Hybrid DTI has sufficient gradient diffusion encoding directions to probe the angular resolution of fiber tracts in grey matter and its multi-shell set-up further probes different (fast to slow) diffusion scales of tissue [31,32]. It can be viewed as a simplification of diffusion spectrum imaging, which probes tissue diffusion properties more comprehensively, but at a significant time penalty [33]. All sequences used the same field of view (FOV) of 64 × 64 mm and matrix size of 128 × 128 for each individual sample to achieve a 500 µm isotropic resolution (Table 1) and afford a geometrical overlay of images without requiring co-registration. For illustration purposes and comparisons to clinical scans, MR images were downsampled to a 2 mm isotropic voxel size in FIJI (https://imagej.net/Fiji, access date 28 September 2020). The data that support the findings of this study are available from the corresponding author upon reasonable request.

### 2.3. Diffusion Image Processing

**Figure 2:**
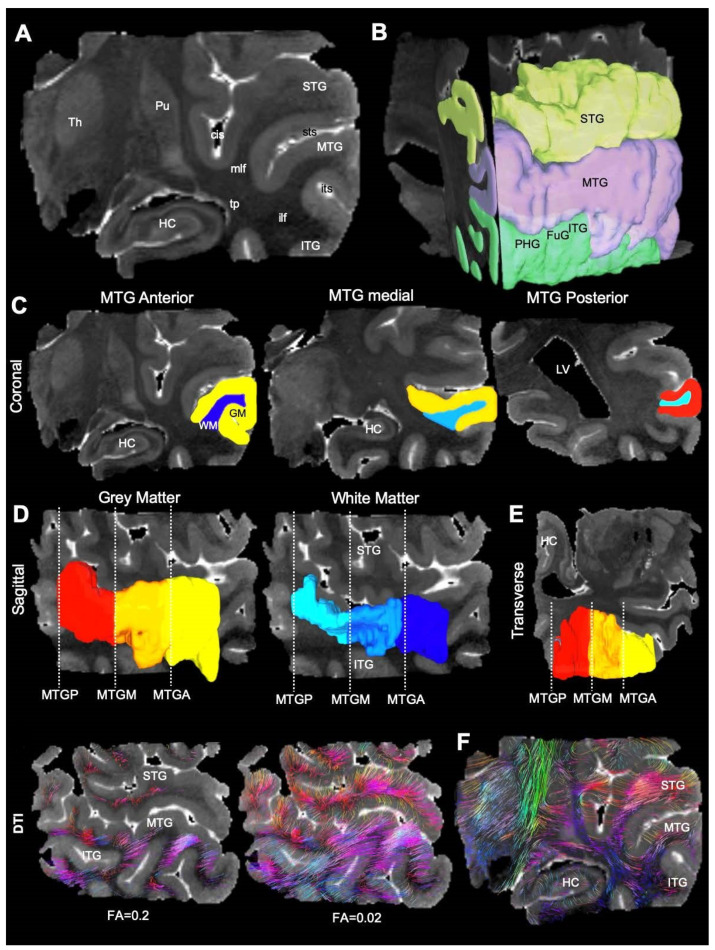
Ex vivo mesoscopic MR imaging of the postmortem non-diseased temporal lobe. (**A**) Coronal view through the temporal lobe reveals its internal anatomy in addition to surface landmarks, such as the superior (sts) and inferior temporal sulci (its). The circular sulcus (cis) was also evident in the coronal plane. The hippocampus, as well as the interior (ITG), medial (MTG), and superior temporal gyrus (STG) are identified in addition to white matter regions consisting of the inferior longitudinal fascicle (ilf), middle longitudinal fascicle (mlf), and the tapetum (tp). The thalamus (Th) and putamen (Pu) are also visible in this plane. (**B**) Segmentation of the ITG, MTG, and STG. Within the ITG, the fusiform gyrus (FuG) and the parahippocampal gyrus (PHG) are identified. (**C**) The MTG is divided into the anterior, medial, and posterior parts. A further distinction is made between the cortical mantle and the white matter within the MTG. (**D**) Three-dimensional rendering of the grey matter of the cortical mantle versus the white matter of the sagittal view plane of the MTG. (**E**) Transverse view of the MTG. (**F**) A high FA value (=0.2) typically used to trace white matter tracts visualizes streamlines confined to the white matter, whereas a lower FA termination value (=0.02) affords tracing of fiber tracts from the white and grey matter. Applying a low FA termination threshold affords the visualization of an extensive network of connections in the temporal lobe.

**Figure 3:**
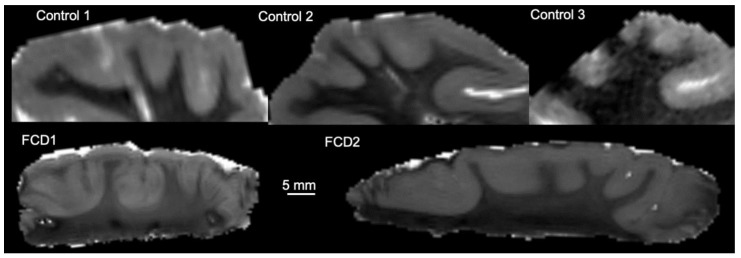
Representative greyscale MR images. Representative mean diffusivity (MD) images of the central slice of the medial temporal gyrus of the two FCD cases, as well as the 3 control postmortem hippocampi.

Diffusion images were processed using DSI studio (http://dsi-studio.labsolver.org, access date 28 September 2020) [34] Maps for individual diffusion metrics, including fractional anisotropy (FA), mean diffusivity (MD), radial diffusivity (RD), and axial diffusivity (AD) were generated and saved as NIfTI-1 files. For surgical specimen, regions of interest (ROIs) were manually drawn on MD images to delineate grey and white matter. For control specimen, whole temporal lobes were scanned to avoid damage to white matter tracts. These scans included the hippocampus and the overlying cortical regions (Figure 2A). On the spin echo DTI datasets, ROIs were drawn to delineate the superior (STG), middle (MTG), and inferior temporal gyri (IFG, Figure 2B). The dentate gyrus (DG), fusiform gyrus (FuG), and parahippocampal gyrus (PHG) were also identified and delineated. Grey and white matter were separated along the anterior-posterior axis of the MTG, also known as Brodmann area 21 (Figure 2C). The MTG was subdivided into the anterior (MTGA), medial (MTGM), and posterior segments (MTGP, Figure 2D). The medial portion of the MTG, which most closely compares to the region and volume of the surgical specimens, was utilized in comparative analysis (Figure 2E, the central slice of the MTG for all samples is presented in Figure 3). Region statistics were generated in DSI studios for each of the ROIs. For each ROI, the following measures were recorded from spin echo DTI datasets: voxel count and volume (mm^3^), as well as the mean of FA, MD, RD, and AD. To account for potential differences in control and surgical sample preparation, absolute diffusion measurements and GM/WM ratios are reported.

### 2.4. Tractography

**Figure 4:**
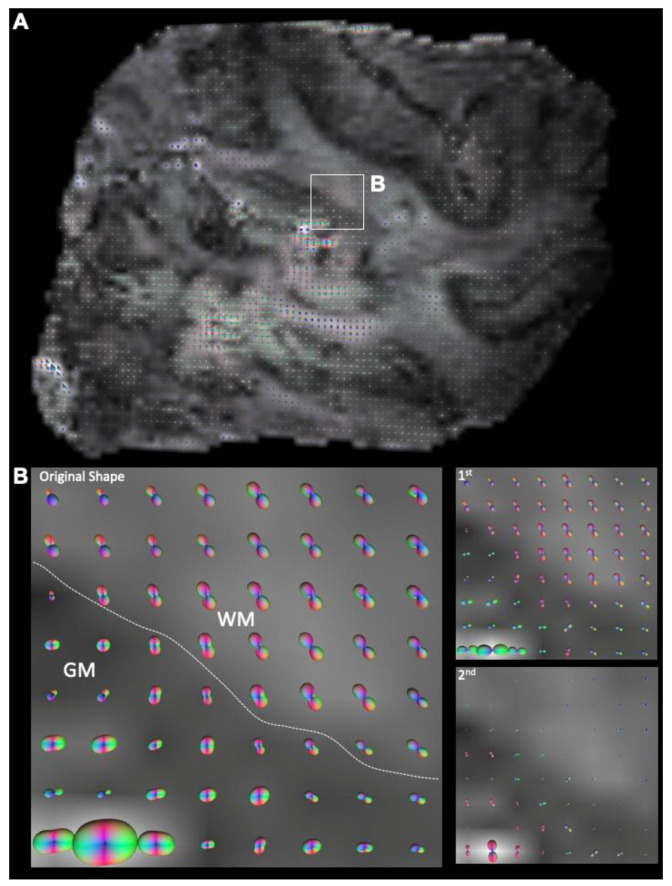
Orientation distribution functions (ODF) of the FCD temporal lobe. (**A**) ODFs displayed for each voxel on transverse slice of the excised temporal lobe of a patient with FCD. (**B**) Contrasting ODF in grey (GM) and white matter (WM) using original, 1st, or 2nd order shape visualizations. A resolution of intersection fibers in the same voxel is evident by comparing the 1st and 2nd order fiber orientation ODFs, especially in GM.

Images were masked to remove background regions to increase reconstruction efficacy in DSI Studio. A model-free reconstruction of the hybrid DTI dataset was performed using a generalized q-sampling imaging (GQI) paradigm [35]. The spin distribution function (SDF), the equivalent measure of orientation distribution function (ODF) for GQI, was computed and visualized the probability of fiber directions contained in each voxel. SDFs were displayed for each voxel (Figure 4A) to determine a separation of intersecting diffusion traces within a single voxel (Figure 4B). Tractography was performed using DSI studio’s local multi-direction deterministic fiber-tracking algorithm for fiber reconstructions, as described previously [21,22,29]. Seeds were randomized (10 seeds/voxel) and positioned at the subvoxel level with all seed orientations considered. A Euler tracing algorithm produced streamlines at a step size of 0.25 mm (half the length of a voxel edge), with a minimum tract length of 1 mm (2× size of voxel edge) and maximum length of 50 mm. As streamlines do not strictly align with each voxel, an estimate to the nearest-neighbor interpolation defined the position of individual streamlines in voxel space. Tracing was terminated at voxels with an FA < 0.02 or a turning angle >60° (Figure 2F). No smoothing was applied. This approach produced robust streamlines in both white and grey matter (Figure 2F The number of streamlines, the mean streamline length and standard deviation, streamline density (streamlines/mm^3^), and streamline volume were recorded for each sample [36].

### 2.5. Histology

Tissue pieces were cut at 50 µm thickness directly on glass slides on a cryostat (Leica) after the sample was cryoprotected in 30% sucrose + 0.5% sodium azide. Sections were washed 3 × 5 min in PBS prior to overnight application of the primary antibodies, notably the pan-neuronal rabbit anti-FOX3 (1:1000, Abcam, ab104225), chicken anti-myelin basic protein (MBP, 1:100, Abcam, ab134018), and the human specific astrocyte mouse anti-SC123 antibody (1:500, Takara Bio, Y40420). Sections were washed 3 × 5 min to remove the primary antibody before application of the appropriate AlexaFluor secondary antibodies (1:500, Molecular Probes) for 1 h. All immunohistochemistry was performed at room temperature (21 °C). After removal of the secondary, DAPI (1:10,000, 5 min) was applied before 3 more washes for 5 min and application of coverslip using Vectashield for fluorescence (Vector Labs). Images were acquired on a M2 Axioimager microscope (Zeiss).

### 2.6. Statistical Analyses

All data were graphed and analyzed in Prism v8.02 (GraphPad Software, San Diego, CA, USA). Control versus FCD data were evaluated for statistical significance (set at *p* < 0.05) using a Mann–Whitney test in case of a direct group comparison or a Kruskall–Wallis test to compare multiple regions followed by a Dunn’s post hoc test.

## 3. Results

### 3.1. Magnetic Resonance (MR)-Histology of the Excised Human Cortex

**Figure 5:**
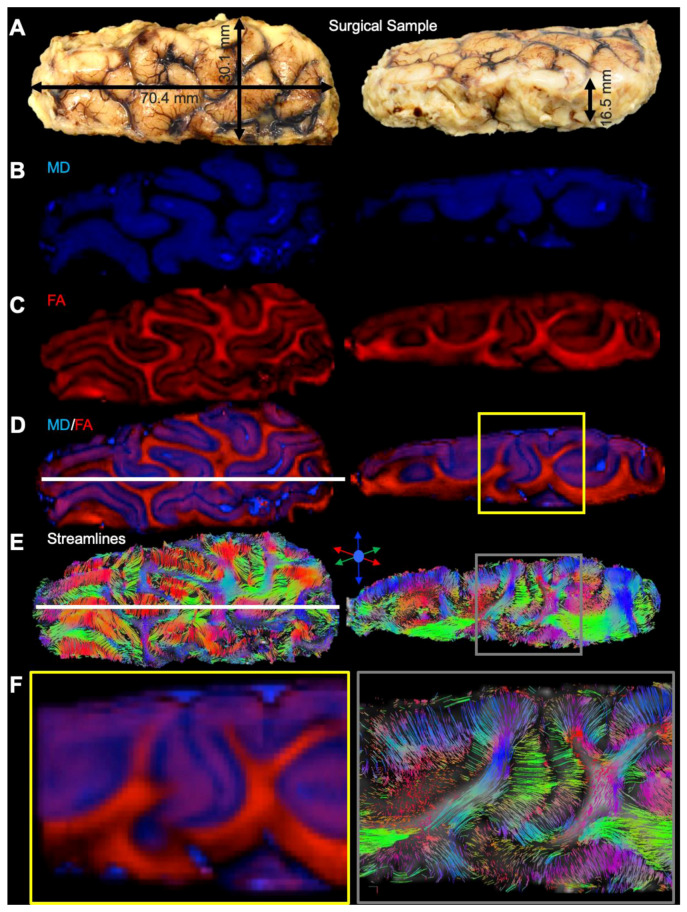
Diffusion tensor imaging (DTI) of surgically excised cortical dysplasia samples. (**A**) Surgically excised samples consisted of the middle temporal gyrus (MTG) and were measured in each dimension in transverse and sagittal plane to define image dimension required for MR imaging. (**B**) The surface identification of gyri was readily visualized in mean diffusion (MD) images, reflecting the high cellularity of the grey matter versus the lack of cellularity in sulci. (**C**) In contrast to MD images, high fractional anisotropy (FA) value were evident in white matter that passed in between gyri. (**D**) Color-coding of MD (blue) and FA (red) images afforded an overlay of both contrasts. A combined image provided a better definition of microenvironments that aided in the segmentation of regions of interest. (**E**) Tractography of these samples further revealed streamlines that connected grey matter through white matter and afforded a unique view of individual gyri. (**F**) A cut-out region of the samples highlights the exquisite detail that defines the cerebral mantle versus the underlying white matter, as well as individual streamlines that fan out from the white into grey matter in individual gyri.

Excised cortical samples were measured to define the imaging dimensions required to visualize the entire length, width, and thickness of samples (Figure 5A). The same measurements were applied to whole temporal lobes obtained from controls (see Table 1 for comparison). The FOV and matrix size were adapted to achieve a 500 µm isotropic resolution. Computation and color-coding of scalar indices from diffusion MRI afforded a view of “grey matter” using MD images (Figure 5B), whereas FA images highlighted “white matter” (Figure 5C). Each contrast provides complimentary information about the tissue microstructure and overlay of both provides a histology-like image that reflects a tissue structure which is not easily discerned on individual contrast images (Figure 5D). The generation of streamlines, probing white matter fiber tracts, and their color-coding to indicate directionality also provide a unique view of tissue structure that neither the MD, nor the FA, image provide (Figure 5E). Fiber tracts in the white matter are very dense, but streamlines fanning out into the grey matter of the cortical mantle are also evident and provide a detailed view of white to grey matter transition of fiber tracts (Figure 5F). Mesoscale diffusion MRI therefore affords an integrated assessment of white and grey matter of cortical tissue.

### 3.2. Mesoscale Diffusion Characteristics of Cortical Dysplasia

**Figure 6:**
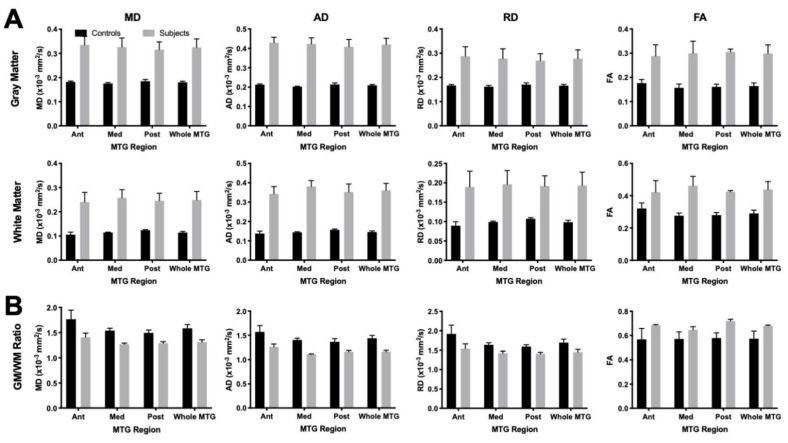
Comparison of scalar indices in temporal lobe regions. (**A**) Diffusion measurements were consistently and significantly (*p* < 0.01) lower for postmortem control compared to surgically excised cortical dysplasia samples, potentially reflecting effects of tissue fixation. (**B**) To provide an intra-sample comparison, grey to white matter ratios were calculated. FCD samples revealed a significantly (*p* < 0.01) lower grey (GM) to white matter (WM) ratio compared to control samples for MD, AD, and RD. For FA, the GM/WM ratio was higher for FCD samples, which reached statistical significance (*p* < 0.01) in the posterior aspect of the MTG.

To quantitatively compare diffusion properties of control and FCD samples, scalar measures were quantified for grey and white matter (Figure 6A), as well as a ratio between both (Figure 6B). Scalar measures in FCD samples were consistently higher for both grey and white matter, potentially reflecting effects of tissue fixation on these measures. The GM/WM ratio reflects relative changes, which should be less affected by tissue fixation. A GM/WM ratio of 1 indicates that both signals are equivalent, whereas higher values (>1) indicate a higher signal in GM and lower values (<1) reveal a higher signal in WM. The GM/WM ratio was consistently higher (*p* < 0.01) for controls on MD, AD, and RD. These results indicate that the difference in GM and WM in FCD cases was less pronounced than in controls. Although the anterior MTG has higher GM/WM ratio for controls compared to FCD, no significant region or interactive effects were found. The higher GM/WM ratio on FA in FCD cases (*p* < 0.01) suggests the same effect, as FCD values are closer to 1 than controls. A reduced GM/WM ratio is potentially a reflection of cortical blurring.

**Figure 7:**
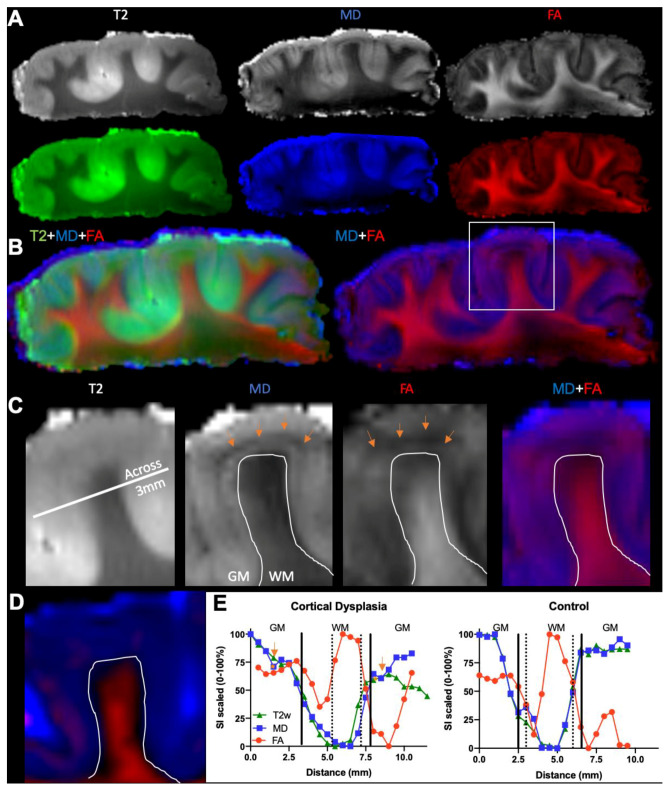
Line profile analysis (LPA). (**A**) LPA was conducted on pseudo-colored red fractional anisotropy (FA) and blue mean diffusivity (MD) images to determine signal intensity variations in the white (WM) to grey matter (GM) transition. (**B**) An overlay of different MR contrast images reveals unique insights into tissue boundaries, such as the grey to white matter transition. (**C**) However, T2-weighted images visually do not add more information than what is provided by MD maps, which complement the information contained in FA maps. Interestingly, a narrow hypointense band was evident in the cortical mantle (orange arrows). This was very pronounced on MD maps, but less so on FA maps and not evident at all on T2-weighted images. These changes were widespread in the FCD sample, although the narrow banding was not consistently present. A straight line across the folium was used for LPA. A high level of FA is evident here in the grey matter of the FCD folium with a high MD signal in the GM/WM transition. (**D**) Control specimen did not exhibit this type of contrast. (**E**) Plotting of signal intensities revealed marked differences in signal intensities in WM, GM,0 and the transition zones between FCD and control folia.

To evaluate cortical blurring, an LPA was performed across and along the length of the gyrus on T2w, MD, and FA images (Figure 7A). Each image provided unique contrast information that highlighted different aspects of the tissue. An overlay of images of an FCD sample revealed a hypointense transition area between the “stem” of the white matter in the gyrus and the grey matter of the cortical mantle that has both an MD and FA signal (Figure 7B). Interestingly, a hypointense band was evident in the grey matter on MD images, but was not evident on T2w images (Figure 7C). A high MD signal and medium level of FA signal is found in grey matter across the gyrus of the FCD sample (Figure 7C), but not the control (Figure 7D). T2w images and their quantitative assessment only poorly reflect cortical blurring, whereas a difference between FA and MD provides a more detailed assessment of tissue abnormalities. Specifically, FA drops markedly at the edge of grey matter before rising sharply again in the stem of the white matter (Figure 7E). This is likely a reflection of white matter organization, where fibers are very aligned at the core as they enter an individual gyrus, but then fan out from that core into grey matter. In the cortical mantle, the tissue is organized in columns that convey a higher FA value. MD is almost completely absent in core white matter, producing a high GM/WM contrast. Along the length of the gyrus, very little MD signal is evident in WM, which contrasts sharply with FA. In the transition zone between WM and GM, a drop in FA is evident. At this juncture, it is expected that MD reveals a sharp boundary to GM. As evident here, there is a slow rise in signal paralleled by FA that is indicative of some cortical blurring. In this case, this blurred white/grey matter junction is approximately 1 mm.

### 3.3. Histological Underpinnings of Grey Matter Abnormalities

**Figure 8:**
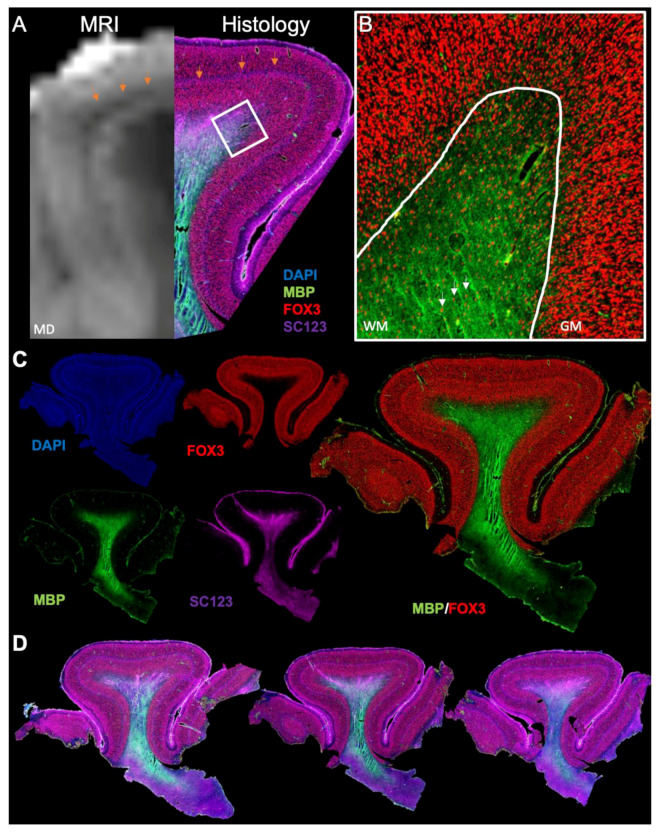
MRI to histology comparison. (**A**) The narrow hypointense band on the T_2_-weighted MR images corresponded to an equal-sized band of cortical layer composition abnormality that is consistent with a tangential migration deficit observed in FCD 1b. Grey to white matter blurring areas were consistent with a lower cellular density in the myelinated (myelin basic protein, MBP) white matter, predominantly astrocytes (SC123). (**B**) However, some interstitial neurons (Fox3) were also evident, but consistent with superficial human neocortex. (**C**) A separation of individual fluorescent channels further highlights abnormal cortical layer composition using the nuclear marker DAPI, as well as the confinement of reactive astrocytes to the myelinated white matter. (**D**) A multi-slice comparison of the folium reveals a consistent pattern of these pathological features, but also highlights the limitations of gaining a more complete view of the pathology throughout the sample and the inability of histology to trace connectivity at the systems scale.

An MRI to histology comparison substantiated the hypointensity in the cortical grey matter (Figure 8A), reflecting an abnormal tangential layer composition at histopathology associated with FCD type 1b. At the WM/GM junction, neurons were also present within the white matter (Figure 8B), with a decreasing density towards the center of the white matter. These interstitial neurons are characteristic of the adult superficial human neocortex. An increased density of these could contribute to the increased MD observed in the LPA. Immunohistochemistry revealed a detailed cellular view of the cortical folium (Figure 8C). The abnormal cell layer is particularly evident using the nuclear DAPI stain, but a bilayer separation of the cortical mantle based on neuronal density is also apparent. Myelin staining was most prominent at the core of the white matter, but was absent as axons fanned out into the cortical mantle. Reactive astrocytes were present throughout the cortical mantel (i.e., grey matter), but were more prominent in the white matter that was contained within the cortical folium. Although immunohistochemistry provides a very detailed view of the cellular composition across multiple slices of the same folia (Figure 8D), it does not provide any indication of connectivity within this folium or with adjacent gyri.

### 3.4. Tractography Reveals Connectivity Changes Associated with Cortical Dysplasia

**Figure 9:**
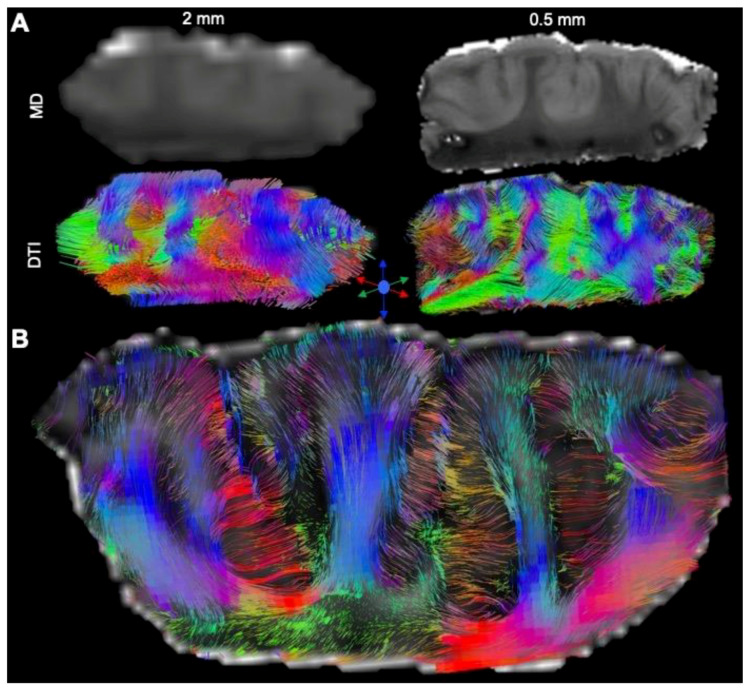
Tractography of cortical dysplasia. (**A**) Downscaling of mesoscale MR images (0.5 mm isotropic) to a typical clinical resolution (2 mm isotropic) demonstrates the inherent limitations of lower-resolution scans to identify and separate individual folia, as well as partial volume effects that compromise a robust delineation of white and grey matter. Partial volume effects are also evident in the tractography at the clinical resolution, especially in terms of distinguishing streamlines entering the grey matter. (**B**) In contrast, an isotropic mesoscale resolution provided a robust tractography through the sample in a coronal view. Branches of white matter feeding into individual gyri before fanning out into the cerebral mantle are readily visualized and traced.

**Figure 10:**
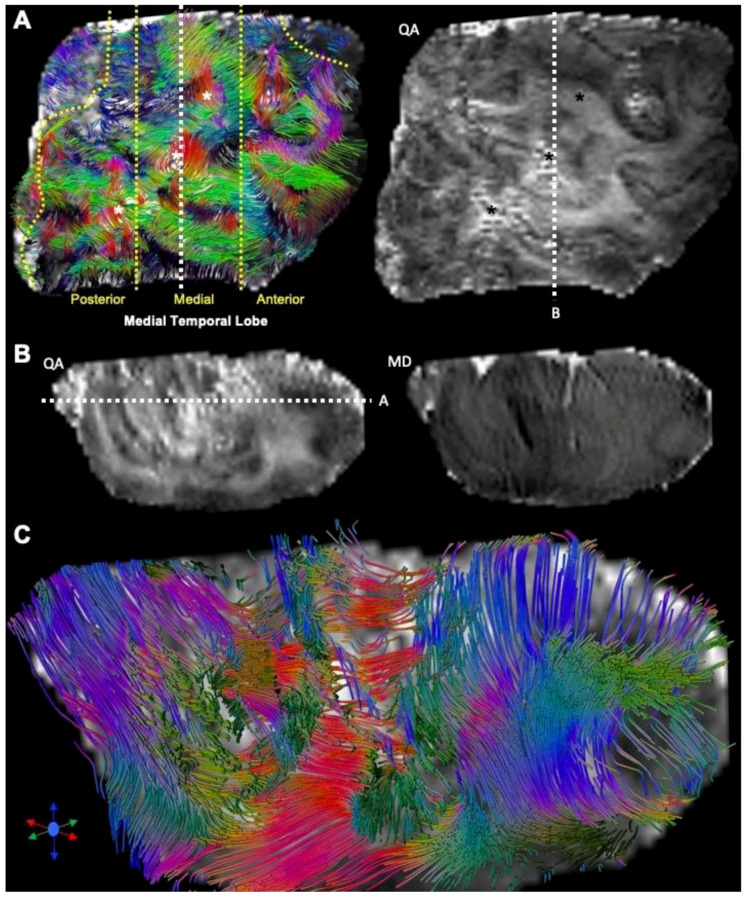
FCD lesions and connectivity. (**A**) A transverse cut of the cortical dysplasia samples afforded a division into the anterior, medial, and posterior portion. High quantitative anisotropy (QA) and disorganized connectivity identified a region in the posterior-medial portion of the medial temporal gyrus (MTG) that is suspected to be a “lesion”. (**B**) A coronal view through the “lesion” locus did not reveal a well-defined gyral structure on the QA or MD image. (**C**) Tractography exposed a disorganized connectivity in this gyrus, with many streamlines uncharacteristically intersecting.

**Figure 11:**
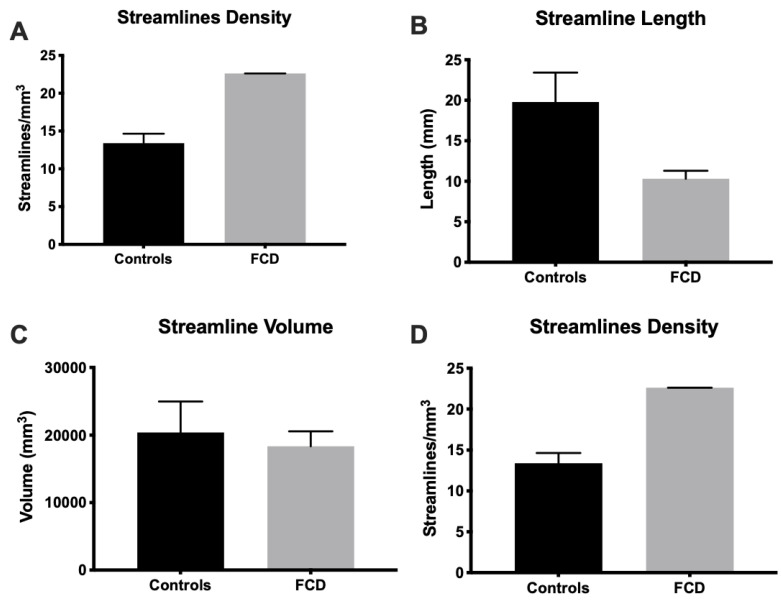
Connectivity difference between control and cortical dysplasia samples. (**A**) A quantitative comparison of regions in the cortical dysplasia samples with those from controls indicated a higher number of streamlines (*p* < 0.05). (**B**) The mean streamline length was longer for control samples (*p* < 0.01). (**C**) However, the volume occupied by streamlines in the MTG was equivalent between controls and cortical dysplasia samples. (**D**) Streamline density in FCD samples (22.6 streamlines/mm^3^) was significantly (*p* < 0.001) higher than control samples (13.4 streamlines/mm^3^).

**Figure 12:**
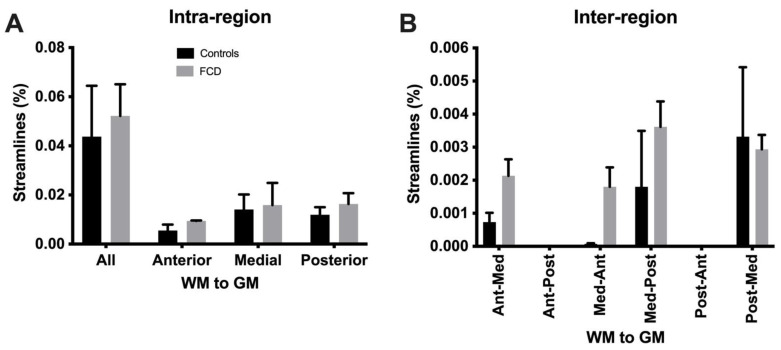
Inter- and intra-regional connectivity in cortical dysplasia. (**A**) Relative white to gray matter connectivity within the MTG did not reveal a significant difference between controls and FCD samples. (**B**) Streamline analysis between white matter in region of the MTG and grey matter in another region (i.e., inter-region connectivity) revealed a significantly (*p* < 0.01) higher connectivity between the anterior to medial MTG for FCD samples when compared to controls, but none of the other connections were significantly different.

DTI at a clinical resolution affords a visualization of streamlines passing through the tissue (Figure 9A). However, at a downsampled 2 mm isotropic voxel size, key anatomical features cannot be visualized (separation of individual folia), whereas a 0.5 mm voxel size readily affords an identification of individual gyri, as well as a separation of the white and grey matter. Lower-resolution tractography produces streamlines that are inconsistent with the anatomical detail visible on the higher resolution tractograms. Mesoscale tractography in these samples therefore afforded a network view of fiber connections that span different gyri and regions (Figure 9B). Tracing of fibers from different regions affords a visualization of connectivity within the MTG and allowed a parcellation of connectivity into its posterior, medial, and anterior aspects (Figure 10A). A “lesion” was evident on the QA image in the posterior-medial aspect of the MTG and affected the corresponding tractography. Within this “lesion” portion of the sample, cortical gyri did not reflect the typical white/grey matter organization observed in other folia (Figure 10B). Compared to adjacent gyri, a disorganized pattern of streamlines was evident within this region (Figure 10C). A quantitative analysis of streamlines revealed significantly more (*p* < 0.05, Figure 11A), but shorter (*p* < 0.05, Figure 11B) streamlines in FCD samples compared to controls. There was no difference in the streamline volume (Figure 11C), but streamline density was significantly higher in FCD samples (*p* < 0.01, Figure 11D). The proportional intra-regional connectivity was equivalent between controls and FCD samples (Figure 12A). By probing more specifically connections between different regions, higher connectivity was evident between the anterior-medial regions (*p* < 0.05) (Figure 12B). Medial-posterior connections in FCD cases were also increased, but due to the small sample size this did not reach statistical significance. It is hence tantalizing to consider if a disorganized and higher connectivity in the medial MTG could represent a characteristic pathological feature of these cases.

## 4. Discussion

A major limitation to our understanding of pediatric epilepsy is the lack of imaging tools for non-invasive subtype classification of FCD lesions and associated aberrant connectivity. We here demonstrate foremost of all that ex vivo mesoscale diffusion MR imaging can detect microstructural defects and tractography can highlight aberrant connectivity in excised surgical epilepsy samples of confirmed cortical dysplasia type I cases. Although extended imaging times are required, these are easily accommodated in an experimental research paradigm and can demonstrate novel QIB targets. Furthermore, our results here demonstrate that fiber tracts surrounding the lesion locus exhibit an aberrant pattern with an overall increase in connectivity, especially between the anterior-medial aspects of the MTG. The cause of this aberrant connectivity remains unclear, but it can be stipulated that it reflects an underlying developmental abnormality in tissue architecture or is the product of peri-lesional re-wiring. Mesoscale resolution also affords a more robust analysis of white/grey matter blurring, with LPA providing a visualization of quantitative changes between the stem of white matter in a cortical gyrus and its transition to grey matter in the cortical mantle. As a 0.5 mm isotropic mesoscale voxel is volumetrically 64x smaller than standard clinical imaging at a 2 mm macroscopic voxel size, this decrease in voxel volume reduces partial volume effects and potentially confounding effects due to signal averaging at the white/grey matter junction. Future work is, nevertheless, needed to perform rigorous comparisons of QIBs derived from clinical imaging protocols and the mesoscale measurements described here.

### 4.1. Ex Vivo MR Imaging as an Analytical Tool in Pediatric Epilepsy

The availability of surgical resections for ex vivo MR imaging offers new opportunities to investigate the pathological basis of epilepsy [29]. The main focus of ex vivo MRI in FCD so far has been to corroborate MR imaging with histological analysis [19,20,37], especially in cases that were classified as MRI-negative. Histological analyses in these cases have helped to identify the lesion locus and allowed an evaluation of the lesion versus peri-lesion area. A very strong T_2_ hyperintensity in the white matter in the lesion locus was associated with FCD IIb and related to dysmyelination [20]. The presence of balloon cells in white matter affecting the myelination process was hypothesized as the putative cause of this histopathological feature. We are not aware of similar investigations using samples of FCD type I. Subtle changes are more difficult to detect on clinical resolution MRI scans and hence can produce an MRI-negative classification [20]. Our study demonstrates that the mesoscale imaging described here has the potential to provide additional value in not only more detailed internal characteristics of the FCD lesions, but also with grey/white matter relationships and associated aberrant connectivity.

Cortical lamination and dysmyelination are two pathological features that affect tissue microstructure and alter the diffusion properties of white and grey matter [38]. Increasingly, diffusion MRI is used to define cortical layer-specific diffusion signals to evaluate lamination on ex vivo samples [39], as well as in vivo in human patients [40]. MD is sensitive to tissue microstructure [41,42], with grey matter here having a higher diffusivity than white matter, whereas FA showed the inverse pattern. FA in the grey matter reflected the columnar organization of the grey matter and axonal projections. In the cortical mantle, thin fiber tracts align to fan in or out of the white matter stem of a single gyrus. An overlay of both types of scans here revealed nuances of tissue microstructure in a single gyrus not readily appreciated on individual scalar images. An LPA across and along the gyrus revealed that the transition between white and grey matter produces a low signal intensity on both MD and FA. Cortical blurring is evident at this junction. An ex vivo T_2_ and FLAIR investigation at 7T attributed cortical blurring with inhomogeneous staining of myelin within the white matter and further suggested that axonal degeneration was also present in these areas [37]. As the mesoscale reduces partial volume effects between both tissue classes and improves their definition, the degree of blurring can be measured more reliably and potentially provide a QIB that can more accurately describe FCD lesions for sub-classification. We propose that an LPA of MD and FA provides an improvement in evaluating microstructural changes that underpin cortical blurring compared to single line analysis of T_1_ or T_2_ signal intensities.

Dysmyelination is known to be associated with an increase in RD, whereas axonal injury is reflected in a decrease in AD [43,44]. However, RD is also affected by axonal diameter and density. All diffusion measures in FCD samples were increased here. This is likely a reflection of tissue fixation [45,46], rather than an absolute difference due to the underlying neurological condition. Indeed, this might be a concern regarding interpretations and inferences of connectivity in postmortem samples. A major challenge for these studies is the sourcing of appropriate controls that are age-matched, but also where postmortem time to fixation and duration of fixation are similar [8,20,37]. Nevertheless, these control measures provide context and relative measures, such as GM/WM ratio, are less affected by absolute differences due to fixation and afford a group comparison. Indeed, an in vivo and ex vivo study of the hippocampus found that the relative measurements were proportional, although there were absolute differences in signal measurements [47]. A decrease in scalar indices and an increase in FA in the GM/WM ratio were observed here. Cortical blurring is the consequence of a higher GM/WM, with the signal in grey and white matter being indistinguishable at a ratio of 1. In vivo FA in frontal or occipital cortex in patients with FCD exhibited an increase, but this was in an absolute measurement that included grey and white matter [15]. These results indicate that improving the spatial resolution of clinical scans could have a major impact on the non-invasive classification of FCD.

### 4.2. Experimental and Interpretation Limitations Inherent to Ex Vivo Mesoscale MR Imaging and Surgical Tissue Samples

Acquiring isotropic mesoscale resolution diffusion MR images is very challenging as small sample movements over protracted imaging time can affect image quality as well as the accuracy of tractography. Tractography at this resolution covering both white and grey matter is also quite different to macroscopic white matter tractography that is typically undertaken in a clinical setting. Not only are there significantly more voxels and streamlines, but also the tractography settings need to be adapted for the mesoscale. A major limitation for this is that tractography paradigms, such as GQI, are designed to terminate (e.g., QA, angular threshold) as fibers enter white matter. To trace connections into grey matter, these termination thresholds become less useful. At present, no paradigm exists to terminate, for instance, fibers as they reach a specific cell layer or to limit tracing between two cell layers [21]. As resolution improves and streamlines visually resemble individual connections, it is further tempting to equate streamlines with individual axons, but this is not the case [48]. From an interpretation perspective, it hence remains unclear what biological components these streamlines really represent. Further comparisons of tractographical results in these samples with histological measures of axonal density and angularity could improve our conceptualization of these streamlines in healthy, as well as diseased, tissues. However, comparing essentially 2D measurements in histology with 3D measurements in MRI poses methodological challenges that have not yet been overcome [49].

A major limitation that emerges from ex vivo diffusion MRI studies on surgically resected tissues relates to how these samples can be analyzed and compared to controls. Comparisons to control subjects are inherently limited by methodological differences in sample preparations (i.e., fixation liquid and time to fixation) that potentially obscure measurement differences [50,51], although they can be helpful to define the normal anatomical context [29]. Especially in pediatric epilepsy cases, a further challenge is to find age-matched controls. Considering rapid changes in brain structures during development, such as myelination during adolescence, it will be difficult to achieve group-wise comparisons. As indicated here, ratiometry could provide a means to use sample inherent tissue features to normalize signal intensities and afford comparisons across multiple samples. Another strategy borrowing from approaches implemented in brain tumor MR imaging [52,53], is to identify “normal” tissue elements in the surgical sample to provide an inherent control measure that would allow interpretation of measurements within the “lesion” and the “peri-lesion” environment [54]. Although these tissue boundaries in resected samples might contain “non-diseased tissues”, surgical trauma could affect measurements in these regions. It would also be unclear to what degree measurements of streamline density in neighboring regions (e.g., ITG versus MTG) could provide normative information about the affected region.

To overcome methodological differences between resected and control samples, the inclusion of different FCD subtypes could provide a quantitative comparison to identify key distinguishing features. The small sample size here serves as an illustration of the utility of mesoscale diffusion MRI in characterizing surgical resections from FCD patients, but also highlights the challenges of drawing conclusions from a small number of cases. We here propose that large cohort studies that incorporate different FCD subtypes are likely more valuable to discern subtype-specific QIB than comparisons to postmortem controls [19,20,37].

The ultimate aim of this endeavor is to provide a robust evidence-based classification of FCD and to improve diagnostic tools that aid in the stratification of patients and their selection for surgical intervention [6].

### 4.3. Mesoscale MR Imaging as a Frontier for Precision Medicine in Pediatric Epilepsy

In vivo imaging of cortical lamination and myelination is advancing rapidly [40,55]. Diffusion MRI currently provides the most sophisticated in vivo imaging tool to define microstructural characteristics of cortical laminae in different regions, as well as the basis to visualize the connectivity between these regions [56]. Improvements in signal acquisition with multi-channel coils and high field 7T human MRI scanners gradually approach the mesoscale to achieve in vivo hybrid DTI [57,58]. Determining changes in connectivity within the MTG therefore can potentially provide novel insights into aberrant axonal projections, but also provide another measure that could stage or differentiate FCD subtypes. For instance, we have shown here that there was an increase in connectivity in FCD type I samples. A hyperconnectivity in FCD IIa is contrasted with a hypoconnectivity in FCD IIb [18] and potentially provides a QIB that could aid in the refinement of FCD subtype classification.

Improving the resolution of diffusion MR and standardization of tractography could provide a greater diagnostic value [29,59]. However, more systematic ex vivo studies and comparison with in vivo imaging results on a larger number of specimens will be required to support the translation of this approach into clinical practice. Using high field 7T clinical MRI scanners will achieve the required resolution, but the time scale required for in vivo imaging [60] will still limit this approach to the pre-operative period. These high-resolution images can be super-imposed in theatre to enhance lower resolution intra-operative scans and improve the surgical procedure [61,62]. The importance of further characterizing FCD lesions using ex vivo diffusion MRI and other modalities [63] therefore has the potential to impact the surgical management of children with epilepsy.

## 5. Conclusions

We here demonstrate that diffusion MR imaging at the mesoscale can provide direct measures of cortical tissue microstructure and afford a delineation of cortical blurring [20,37]. The use of ex vivo MR imaging of surgically excised tissue provides a unique opportunity to investigate and define QIBs that aid in the non-invasive subtype classification of FCD [6,19,20,37]. Although epilepsy is widely considered a connectivity disorder [64], macroscopic tractography is insufficient to reveal connectivity changes within grey matter [22,29]. We here demonstrated that tractography at the mesoscale can provide complimentary information about intra- and inter-regional connectivity that might facilitate the delineation of the full extent of a lesion underlying the seizure activity, as well as any associated networks.

## Figures and Tables

**Table 1:** Physical dimensions of focal cortical dysplasia (FCD) imaging specimen. Voxel size is 0.5 × 0.5 × 0.5 mm = 0.125 mm^3^ voxel volume.

Sample	Length (mm)	Width (mm)	Thickness (mm)	Volume (mm^3^)	Voxels
FCD—A	47.6	35.5	19.1	21,765	174,121
FCD—B	70.4	30.1	16.5	22,553	180,423
Control—A	58.6	50.8	60.9	143,365	1,146,920
Control—B	56.4	57.5	58.2	145,200	1,161,600
Control—C	62.9	42.6	64.3	123,095	984,759

## Data Availability

Data available on request due to privacy restrictions. The data presented in this study are available on request from the corresponding author.
